# Editorial: Enlarged perivascular spaces: etiology and significance

**DOI:** 10.3389/fnins.2023.1321691

**Published:** 2023-12-15

**Authors:** Michele Cavallari, Florian Dubost, Charles R. G. Guttmann, Christopher W. Lee-Messer

**Affiliations:** ^1^Department of Radiology, Center for Neurological Imaging, Brigham and Women's Hospital and Harvard Medical School, Boston, MA, United States; ^2^Department of Neurology, Stanford University, Stanford, CA, United States

**Keywords:** enlarged perivascular spaces (ePVS), magnetic resonance imaging (MRI), diffusion tensor imaging (DTI), Alzheimer's disease (AD), cerebral amyloid angiopathy (CAA), obstructive sleep apnea (OSA), glymphatic clearance, traumatic brain injury (TBI)

Enlarged perivascular spaces (EPVS), also known as Virchow-Robin spaces, are fluid-filled cavities surrounding the brain's small vessels and visible on magnetic resonance imaging (MRI). Multiple mechanisms have been suggested as possible causes of the enlargement of perivascular spaces. The systematic review by Okar et al. provides a comprehensive summary of the different mechanisms that may contribute to the development of EPVS. Vascular abnormalities linked to hypertension and cerebral small vessel disease, as well as brain atrophy associated with neurocognitive disorders, have traditionally been considered relevant factors for the development of EPVS. In addition to these conditions, the recent discovery of the glymphatic pathway of clearance has also been linked to the etiology of EPVS.

The glymphatic pathway is a key mechanism of clearance of potentially neurotoxic proteins, such as beta-amyloid, tau, and alpha-synuclein, which are implicated in the pathobiology of neurodegenerative diseases, including Alzheimer's disease, frontotemporal dementia, traumatic brain injury, and Parkinson's disease. Since its discovery in 2013 (Xie et al., 2013), the relevance of the glymphatic pathway of clearance has extended to other conditions, as validated by the two original research articles on obstructive sleep apnea (OSA) (Roy et al.) and cerebral amyloid angiopathy (Lui et al.) included in this Research Topic. The observed glymphatic dysfunction in patients with OSA is of particular interest because the mechanism of glymphatic clearance has been first described during deep sleep (Xie et al., 2013). Given that OSA is associated with an increased risk of Alzheimer's disease, the glymphatic dysfunction observed in these patients by Roy et al. may account for their heightened risk. This interesting finding should be further explored in longitudinal studies that consider cognitive outcomes. Future studies may also help clarify the temporal dynamics of the glymphatic dysfunction observed in patients with OSA across the sleep-wake cycle.

The development and validation of MRI methods to assess the glymphatic pathway of clearance is an area of active research. The perivascular space is a crucial node of the glymphatic pathway. EPVS on MRI have recently emerged as a proxy for glymphatic dysfunction. This Research Topic also includes original research papers using different MRI measures of glymphatic clearance, such as diffusion along the perivascular space (DTI-ALPS) in patients with OSA and contrast-enhanced MRI in an animal model of traumatic brain injury (Gu et al.; Roy et al.). While more invasive imaging methods in animal models provide an insight into the dynamic aspect of the glymphatic clearance, emerging automated methods, such as DTI-ALPS and automated segmentation methods for EPVS (Okar et al.), provide an opportunity for quantitative assessment on large-scale and legacy MRI data. One limitation to the applicability of those MRI measures is linked to the scarcity of longitudinal studies. The systematic review by Okar et al. provides an essential summary of the limited number of studies that assessed the temporal evolution of EPVS.

MRI measures of glymphatic clearance represent promising biomarkers applicable to several highly prevalent neurodegenerative conditions characterized by the brain accumulation of neurotoxic proteins. Those MRI measures hold promise as early biomarkers of impaired clearance before the accumulation of neurotoxic proteins may trigger other mechanisms that are difficult to control, such as neuroinflammation and neurodegeneration. There is growing evidence that the glymphatic clearance can be modulated, for instance by focused ultrasound (Ye et al., 2023). MRI measures of glymphatic clearance may be useful in monitoring the effectiveness of focused ultrasound and similar interventions targeting the glymphatic pathway of clearance that may become available in the future.

## Author contributions

MC: Writing—original draft. FD: Writing—review & editing. CG: Writing—review & editing. CL-M: Writing—review & editing.
